# Supplementary material for: Cholesin mRNA Expression in Human Intestinal, Liver, and Adipose Tissues
Source: Nutrients. 2025 Feb 8;17(4):619. doi: 10.3390/nu17040619 (PMC11858417; doi:10.3390/nu17040619)
Supplement: Supplementary file 1 [file nutrients-17-00619-s001.zip › Gilliam-Vigh et al_cholesin_supplementary repository.pdf]

Supplementary Repository

Location	Relative difference in NPC1L1 mRNA expression between duodenum and other locations in the small intestine		
CTRL	Fold difference (95% CI)	*P*-value	Adjusted *P*-value
Treitz	0.93 (0.62 - 1.40)	0.71	0.87
3	0.91 (0.54 - 1.52)	0.70	0.86
4	0.82 (0.56 - 1.19)	0.27	0.51
5	0.68 (0.42 - 1.11)	0.12	0.34
6	0.77 (0.52 - 1.13)	0.17	0.44
7	0.71 (0.48 - 1.06)	0.09	0.30
8	0.74 (0.49 - 1.12)	0.14	0.39
9	0.78 (0.46 - 1.32)	0.34	0.62
Ileocecal	-	-	-
T2D	Fold difference (95% CI)	*P*-value	Adjusted *P*-value
Treitz	0.84 (0.55 - 1.29)	0.41	0.72
3	0.72 (0.42 - 1.25)	0.23	0.47
4	1.00 (0.67 - 1.49)	0.99	0.99
5	0.85 (0.52 - 1.40)	0.52	0.82
6	0.88 (0.59 - 1.31)	0.51	0.82
7	0.98 (0.65 - 1.47)	0.90	0.96
8	0.70 (0.45 - 1.07)	0.09	0.30
9	0.48 (0.28 - 0.83)	0.01	0.06
Ileocecal	-	-	-

Table S1. Fold difference in NPC1L1 mRNA expression in duodenum (reference) vs other small intestinal locations of healthy controls (CRTL) and patients with type 2 diabetes (T2D). *P*-values were adjusted for multiple testing using the method of Benjamini and Hochberg which controls the false discovery rate. CI, confidence interval.

Location	Relative difference in NPC1L1 mRNA expression between rectum and other locations in the large intestine.		
CTRL	Fold difference (95% CI)	*P*-value	Adjusted *P*-value
Cecum	0.69 (0.46 - 1.06)	0.08	0.28
Ascending colon	0.74 (0.46 - 1.22)	0.22	0.47
Transverse colon	0.64 (0.39 - 1.04)	0.07	0.23
Descending colon	0.77 (0.49 - 1.20)	0.23	0.47
Sigmoid colon	0.86 (0.47 - 1.60)	0.62	0.82
T2D	Fold difference (95% CI)	*P*-value	Adjusted *P*-value
Cecum	0.86 (0.54 - 1.37)	0.52	0.82
Ascending colon	0.95 (0.56 - 1.61)	0.83	0.94
Transverse colon	0.93 (0.55 - 1.57)	0.76	0.91
Descending colon	0.99 (0.60 - 1.61)	0.95	0.97
Sigmoid colon	1.18 (0.61 - 2.30)	0.60	0.82

Table S2. Fold difference in NPC1L1 mRNA expression in rectum (reference) vs other large intestinal locations of healthy controls (CRTL) and patients with type 2 diabetes (T2D). *P*-values were adjusted for multiple testing using the method of Benjamini and Hochberg which controls the false discovery rate. CI. confidence interval.

Locations	Relative difference in NPC1L1 mRNA expression between patients with type 2 diabetes and healthy controls		
Small intestine	Fold difference (95% CI)	*P*-value	Adjusted *P*-value
Duodenum	0.91 (0.63 - 1.32)	0.62	0.82
Treitz	0.92 (0.63 - 1.35)	0.67	0.85
3	0.93 (0.72 - 1.20)	0.55	0.82
4	0.98 (0.83 - 1.17)	0.82	0.94
5	0.90 (0.79 - 1.03)	0.12	0.34
6	0.97 (0.76 - 1.24)	0.81	0.94
7	0.56 (0.33 - 0.95)	0.03	0.14
8	0.19 (0.07 - 0.50)	1.92×10^{-3}	0.01
9	0.15 (0.02 - 1.20)	0.07	0.24
Ileocecal	-	-	-
Large intestine	Fold difference (95% CI)	*P*-value	Adjusted *P*-value
Cecum	1.44 (0.33 - 6.25)	0.59	0.82
Ascending colon	0.85 (0.55 - 1.31)	0.44	0.74
Transverse colon	1.14 (0.73 - 1.76)	0.55	0.82
Descending colon	0.84 (0.61 - 1.16)	0.27	0.51
Sigmoid colon	0.89 (0.36 - 2.18)	0.79	0.92
Rectum	1.24 (0.53 - 2.93)	0.60	0.82

Table S3. Estimated fold-difference in NPC1L1 mRNA expression in the intestines of healthy controls vs type 2 diabetes. *P*-values were adjusted for multiple testing using the method of Benjamini and Hochberg which controls the false discovery rate. No significant differences between patients with T2D and the group of healthy controls were found after adjusting for multiple testing. CI, confidence interval.

Location	Relative difference in NPC1L1 mRNA expression for RYGB operated individuals		
CTRL	Fold difference (95% CI)	P-value	Adjusted P-value
Alimentary limb	0.66 (0.56 - 0.78)	5.03×10^{-5}	3.20×10^{-3}
Biliopancreatic limb	1.25 (1.02 - 1.54)	0.04	0.15
Common channel	1.02 (0.71 - 1.47)	0.90	0.96

Table S4. Fold difference in NPC1L1 mRNA expression in the intestine post RYGB operation vs before operation. P-values were adjusted for multiple testing using the method of Benjamini and Hochberg which controls the false discovery rate. CI, confidence interval.

	Relative difference in NPC1L1 mRNA expression between individual with obesity and lean controls		
	Mean (95% CI mean)	*P*-value	Adjusted *P*-value
Lean<br>Obese	12.61 (10.20 – 15.03)<br>10.76 (9.27 – 12.25)	0.20	0.45

Table S5. Estimated fold-difference in NPC1L1 mRNA expression in the liver of lean individuals vs obese individuals. *P*-values were adjusted for multiple testing using the method of Benjamini and Hochberg which controls the false discovery rate. No significant differences between individuals with obesity and the group of healthy controls were found after adjusting for multiple testing. CI, confidence interval.

Location	Relative difference in Cholesin mRNA expression between duodenum and other locations in the small intestine		
CTRL	Fold difference (95% CI)	*P*-value	Adjusted *P*-value
Treitz	0.83 (0.71 – 0.98)	0.03	0.13
3	0.66 (0.52 – 0.83)	1.39×10^{-3}	0.01
4	0.68 (0.54 – 0.87)	3.97×10^{-3}	0.02
5	0.65 (0.51 – 0.84)	2.10×10^{-3}	0.01
6	0.67 (0.55 – 0.83)	8.72×10^{-4}	0.01
7	0.77 (0.63 – 0.95)	0.2	0.08
8	0.68 (0.54 – 0.86)	2.74×10^{-3}	0.02
9	0.86 (0.66 – 1.14)	0.28	0.52
Ileocecal	0.84 (0.64 – 1.10)	0.20	0.45
T2D	Fold difference (95% CI)	*P*-value	Adjusted *P*-value
Treitz	0.82 (0.69 – 0.98)	0.03	0.13
3	0.59 (0.47 – 0.76)	3.56×10^{-4}	4.86×10^{-3}
4	0.71 (0.55 – 0.92)	0.01	0.06
5	0.69 (0.53 – 0.89)	7.74×10^{-3}	0.04
6	0.63 (0.51 – 0.78)	1.92×10^{-4}	3.20×10^{-3}
7	0.63 (0.51 – 0.77)	1.70×10^{-4}	3.20×10^{-3}
8	0.59 (0.46 – 0.75)	1.91×10^{-4}	3.20×10^{-3}
9	0.60 (0.45 – 0.80)	1.27×10^{-3}	0.01
Ileocecal	1.12 (0.82 – 1.54)	0.44	0.74

Table S6. Fold difference in Cholesin mRNA expression in duodenum (reference) vs other small intestinal locations of healthy controls (CRTL) and patients with type 2 diabetes (T2D). *P*-values were adjusted for multiple testing using the method of Benjamini and Hochberg which controls the false discovery rate. CI, confidence interval.

Location	Relative difference in Cholesin mRNA expression between rectum and other locations in the large intestine.		
CTRL	Fold difference (95% CI)	*P*-value	Adjusted *P*-value
Cecum	0.87 (0.69 – 1.10)	0.21	0.46
Ascending colon	0.98 (0.80 – 1.19)	0.79	0.92
Transverse colon	0.98 (0.80 – 1.21)	0.87	0.95
Descending colon	0.91 (0.79 – 1.05)	0.19	0.45
Sigmoid colon	0.91 (0.77 – 1.07)	0.24	0.48
T2D	Fold difference (95% CI)	*P*-value	Adjusted *P*-value
Cecum	1.04 (0.81 – 1.33)	0.76	0.91
Ascending colon	1.04 (0.84 – 1.28)	0.70	0.86
Transverse colon	1.09 (0.87 – 1.36)	0.43	0.73
Descending colon	1.18 (1.00 – 1.38)	0.05	0.18
Sigmoid colon	1.16 (0.97 – 1.40)	0.10	0.31

Table S7. Fold difference in Cholesin mRNA expression in rectum (reference) vs other large intestinal locations of healthy controls (CRTL) and patients with type 2 diabetes (T2D). *P*-values were adjusted for multiple testing using the method of Benjamini and Hochberg which controls the false discovery rate. CI, confidence interval.

Locations	Relative difference in Cholesin mRNA expression between patients with type 2 diabetes and healthy controls		
Small intestine	Fold difference (95% CI)	*P*-value	Adjusted *P*-value
Duodenum	0.97 (0.75 – 1.27)	0.84	0.94
Treitz	0.99 (0.72 – 1.36)	0.95	0.97
3	1.08 (0.77 – 1.50)	0.65	0.84
4	0.94 (0.72 – 1.22)	0.62	0.82
5	0.92 (0.71 – 1.20)	0.52	0.82
6	1.05 (0.77 – 1.44)	0.73	0.89
7	1.21 (0.91 – 1.60)	0.18	0.45
8	1.13 (0.81 – 1.57)	0.47	0.78
9	1.41 (1.00 – 1.99)	0.05	0.19
Ileocecal	0.73 (0.46 – 1.17)	0.18	0.44
Large intestine	Fold difference (95% CI)	*P*-value	Adjusted *P*-value
Cecum	1.00 (0.76 – 1.33)	0.99	0.99
Ascending colon	0.89 (0.67 – 1.18)	0.41	0.72
Transverse colon	0.93 (0.73 – 1.18)	0.53	0.82
Descending colon	1.08 (0.83 – 1.40)	0.54	0.82
Sigmoid colon	1.07 (0.82 – 1.40)	0.60	0.82
Rectum	0.84 (0.64 – 1.10)	0.19	0.45

Table S8. Estimated fold-difference in Cholesin mRNA expression in the intestines of healthy controls vs type 2 diabetes. *P*-values were adjusted for multiple testing using the method of Benjamini and Hochberg which controls the false discovery rate. No significant differences between patients with T2D and the group of healthy controls were found after adjusting for multiple testing. CI, confidence interval.

Location	Relative difference in Cholesin mRNA expression for RYGB operated individuals		
CTRL	Fold difference (95% CI)	*P*-value	Adjusted *P*-value
Alimentary limb	0.87 (0.69 – 1.10)	0.21	0.46
Biliopancreatic limb	0.98 (0.80 – 1.19)	0.79	0.92
Common channel	0.98 (0.80 – 1.21)	0.87	0.95

Table S9. Fold difference in Cholesin mRNA expression in the intestine post RYGB operation vs before operation. *P*-values were adjusted for multiple testing using the method of Benjamini and Hochberg which controls the false discovery rate. CI, confidence interval.

	Relative difference in Cholesin mRNA expression between individual with obesity and lean controls		
	Mean (95% CI mean)	*P*-value	Adjusted *P*-value
Lean<br><br>Obese	3.25 (2.90 – 3.60)<br><br>3.22 (2.73 – 3.71)	0.92	0.96

Table S10. Estimated fold-difference in Cholesin mRNA expression in the liver of lean individuals vs obese individuals. *P*-values were adjusted for multiple testing using the method of Benjamini and Hochberg which controls the false discovery rate. No significant differences between individuals with obesity and the group of healthy controls were found after adjusting for multiple testing. CI, confidence interval.

Location	Relative difference in GPR146 mRNA expression between duodenum and other locations in the small intestine		
CTRL	Fold difference (95% CI)	*P*-value	Adjusted *P*-value
Treitz	0.93 (0.62 - 1.40)	0.71	0.87
3	0.91 (0.54 - 1.52)	0.70	0.86
4	0.82 (0.56 - 1.19)	0.27	0.51
5	0.68 (0.42 - 1.11)	0.12	0.34
6	0.77 (0.52 - 1.13)	0.17	0.44
7	0.71 (0.48 - 1.06)	0.09	0.30
8	0.74 (0.49 - 1.12)	0.14	0.39
9	0.78 (0.46 - 1.32)	0.34	0.62
Ileocecal	-	-	-
T2D	Fold difference (95% CI)	*P*-value	Adjusted *P*-value
Treitz	0.84 (0.55 - 1.29)	0.41	0.72
3	0.72 (0.42 - 1.25)	0.23	0.47
4	1.00 (0.67 - 1.49)	0.99	0.99
5	0.85 (0.52 - 1.40)	0.52	0.82
6	0.88 (0.59 - 1.31)	0.51	0.82
7	0.98 (0.65 - 1.47)	0.90	0.96
8	0.70 (0.45 - 1.07)	0.09	0.30
9	0.48 (0.28 - 0.83)	0.01	0.06
Ileocecal	-	-	-

Table S11. Fold difference in GPR146 mRNA expression in duodenum (reference) vs other small intestinal locations of healthy controls (CRTL) and patients with type 2 diabetes (T2D). *P*-values were adjusted for multiple testing using the method of Benjamini and Hochberg which controls the false discovery rate. CI, confidence interval.

Location	Relative difference in GPR146 mRNA expression between rectum and other locations in the large intestine.		
CTRL	Fold difference (95% CI)	*P*-value	Adjusted *P*-value
Cecum	0.69 (0.46 - 1.06)	0.08	0.28
Ascending colon	0.74 (0.46 - 1.22)	0.22	0.47
Transverse colon	0.64 (0.39 - 1.04)	0.07	0.23
Descending colon	0.77 (0.49 - 1.20)	0.23	0.47
Sigmoid colon	0.86 (0.47 - 1.60)	0.62	0.82
T2D	Fold difference (95% CI)	*P*-value	Adjusted *P*-value
Cecum	0.86 (0.54 - 1.37)	0.52	0.82
Ascending colon	0.95 (0.56 - 1.61)	0.83	0.94
Transverse colon	0.93 (0.55 - 1.57)	0.76	0.91
Descending colon	0.99 (0.60 - 1.61)	0.95	0.97
Sigmoid colon	1.18 (0.61 - 2.30)	0.60	0.82

Table S12. Fold difference in GPR146 mRNA expression in rectum (reference) vs other large intestinal locations of healthy controls (CRTL) and patients with type 2 diabetes (T2D). *P*-values were adjusted for multiple testing using the method of Benjamini and Hochberg which controls the false discovery rate. CI, confidence interval.

Locations	Relative difference in GPR146 mRNA expression between patients with type 2 diabetes and healthy controls		
Small intestine	Fold difference (95% CI)	*P*-value	Adjusted *P*-value
Duodenum	1.19 (0.88 - 1.61)	0.24	0.48
Treitz	1.32 (0.81 - 2.14)	0.25	0.49
3	1.49 (0.79 - 2.82)	0.21	0.46
4	0.97 (0.65 - 1.46)	0.89	0.96
5	0.95 (0.49 - 1.81)	0.86	0.95
6	1.04 (0.70 - 1.54)	0.83	0.94
7	0.87 (0.52 - 1.47)	0.59	0.82
8	1.26 (0.74 - 2.14)	0.37	0.67
9	1.94 (1.05 - 3.58)	0.03	0.14
Ileocecal	-	-	-
Large intestine	Fold difference (95% CI)	*P*-value	Adjusted *P*-value
Cecum	1.00 (0.76 – 1.33)	0.99	0.99
Ascending colon	0.89 (0.67 – 1.18)	0.41	0.72
Transverse colon	0.93 (0.73 – 1.18)	0.53	0.82
Descending colon	1.08 (0.83 – 1.40)	0.54	0.82
Sigmoid colon	1.07 (0.82 – 1.40)	0.60	0.82
Rectum	0.84 (0.64 – 1.10)	0.19	0.45

Table S13. Estimated fold-difference in GPR146 mRNA expression in the intestines of healthy controls vs type 2 diabetes. *P*-values were adjusted for multiple testing using the method of Benjamini and Hochberg which controls the false discovery rate. No significant differences between patients with T2D and the group of healthy controls were found after adjusting for multiple testing. CI, confidence interval.

Location	Relative difference in GPR146 mRNA expression for RYGB operated individuals		
CTRL	Fold difference (95% CI)	*P*-value	Adjusted *P*-value
Alimentary limb	1.69 (1.11 - 2.59)	0.02	0.09
Biliopancreatic limb	1.34 (0.93 - 1.94)	0.11	0.31
Common channel	1.31 (0.90 - 1.92)	0.15	0.39

Table S14. Fold difference in GPR146 mRNA expression in the intestine post RYGB operation vs before operation. *P*-values were adjusted for multiple testing using the method of Benjamini and Hochberg which controls the false discovery rate. CI, confidence interval.

	Relative difference in GPR146 mRNA expression between individual with obesity and lean controls		
	Mean (95% CI mean)	*P*-value	Adjusted *P*-value
Lean<br><br>Obese	5.95 (5.25 – 6.65)<br><br>5.66 (4.68 – 6.64)	0.60	0.82

Table S15. Estimated fold-difference in GPR146 mRNA expression in the liver of lean individuals vs obese individuals. *P*-values were adjusted for multiple testing using the method of Benjamini and Hochberg which controls the false discovery rate. No significant differences between individuals with obesity and the group of healthy controls were found after adjusting for multiple testing. CI, confidence interval.
